# SLE patients and autoantibody-positive healthy individuals display unique cytokine profiles: shared features of inflammation as well as select features of immunosuppression in autoantibody-positive healthy individuals

**DOI:** 10.1186/ar3960

**Published:** 2012-09-27

**Authors:** LL Ritterhouse, HT Maecker, CG Fathman, JT Merrill, JM Guthridge, JA James

**Affiliations:** 1 Oklahoma Medical Research Foundation, Oklahoma City, OK, USA; 2 University of Oklahoma Health Sciences Center, Oklahoma City, OK, USA; 3 Stanford University School of Medicine, Stanford, CA, USA

## Introduction

Antinuclear antibodies can be detected in up to 25% of the population; however, only 5 to 7% are afflicted with an autoimmune disease. Therefore, many individuals are capable of mitigating the presence of autoantibodies and avoiding clinical disease. The objective of this study was to investigate unique features of SLE patients compared with autoantibody-positive (aAb^+^) healthy immune systems and to examine roles that cytokines play in maintaining immune equilibrium in ANA-positive healthy individuals.

## Methods

Individuals (*n *= 790) were screened by multiplex, bead-based assays for autoantibodies against: dsDNA, chromatin, ribosomal P, SS-A/Ro, SS-B/La, Sm, Sm/RNP, RNP, SCL-70, Jo-1, and centromere B. Sera from aAb^+ ^individuals, matched aAb^- ^controls and SLE patients were tested for 52 cytokines using multiplex bead-based assays and ELISAs. Hierarchical clustering was performed and Kruskal-Wallis tests with Dunn's multiple comparisons were compared including a false discovery rate. Immune cell phenotyping and phospho-flow cytometry was performed on peripheral blood mononuclear cells from aAb^+ ^and aAb^- ^healthy individuals. Unpaired *t *tests and Mann-Whitney tests were performed.

## Results

Of the screened individuals, 57 individuals were positive for at least one autoantibody (7.2%), with 33.3% being Native American, 57.9% European American, 8.8% African American, and 89.5% female. European American aAb^+ ^healthy individuals (*n *= 31) and matched aAb^- ^healthy controls and SLE patients were selected for further analysis. While aAb^+ ^healthy individuals displayed some similar cytokine patterns to SLE patients, they also displayed a suppressed cytokine profile that included decreased T-cell cytokines (IFNγ (*P *< 0.05), IL-5 (*P *< 0.05), IL-17F (*P *< 0.01)), decreased B-lymphocyte stimulator levels (*P *< 0.05), and increased IL-1 receptor antagonist levels (*P *< 0.001). An increased percentage of B cells was found in aAb^+ ^healthy individuals compared with aAb^- ^healthy controls (*P *= 0.039) that consisted of an increased percentage of memory B cells (*P *= 0.034) and a decreased percentage of transitional B cells (*P *= 0.028). Compared with aAb^- ^healthy controls, B cells from aAb^+ ^healthy individuals showed significantly increased pSTAT1 and pSTAT3 in response to IFNα stimulation (*P *= 0.037 and *P *= 0.040), increased pSTAT1 in response to IFNγ stimulation (*P *= 0.018), and increased pSTAT3 in response to IL-21 stimulation (*P *= 0.041). CD4^+ ^T cells from aAb^+ ^healthy individuals showed decreased pSTAT3 in response to IFNγ and IL-2 stimulation (*P *= 0.018 and *P *= 0.005). IFNα, IFNγ and IL-6 stimulation significantly increased pSTAT signaling in monocytes from aAb^+ ^healthy individuals.

## Conclusion

Although aAb^+ ^healthy individuals exhibit features of inflammation and loss of immune tolerance, they are capable of suppressing these responses by regulatory mechanisms probably no longer functional in patients with autoimmune disease.

